# Effect of GSTM1-Polymorphism on Disease Progression and Oxidative Stress in HIV Infection: Modulation by HIV/HCV Co-Infection and Alcohol Consumption

**DOI:** 10.4172/2155-6113.1000237

**Published:** 2013-08-31

**Authors:** Mary Parsons, Adriana Campa, Shenghan Lai, Yinghui Li, Janet Diaz Martinez, Jorge Murillo, Pedro Greer, Sabrina Sales Martinez, Marianna K Baum

**Affiliations:** 1 R. Stempel College of Public Health and Social Work, Florida International University, Miami, FL, USA; 2 School of Medicine, Johns Hopkins University, Baltimore, Maryland, USA; 3 Herbert Werheim College of Medicine, Florida International University, Miami, FL, USA

**Keywords:** GSTM-1 polymorphism, HIV disease progression, Oxidative stress, HIV/HCV co-infection, Alcohol consumption

## Abstract

**Objective:**

To examine the effects of GSTM1 null-allele polymorphism on oxidative stress and disease progression in HIV infected and HIV/hepatitis C (HCV) co-infected adults.

**Methods:**

HIV-infected and HIV/HCV co-infected participants aged 40–60 years old with CD4 cell count >350 cells/ µl, were recruited. GSTM1 genotype was determined by quantitative PCR. Oxidative stress (mitochondrial 8-oxo-2’-deoxyguanosine [8-oxo-dG], malondialdehyde [MDA], oxidized glutathione and Complexes I and IV), apoptosis and HIV disease (CD4 count and viral load) markers were measured. Gene copies were not quantified, thus the Hardy-Weinberg formula was not applicable.

**Results:**

Of the 129 HIV-infected participants, 58 were HIV/HCV co-infected. GSTM1 occurred in 66% (62/94) in those of African descent, and 33% (11/33) of the Caucasians. Those with GSTM1 coding for the functional antioxidant enzyme Glutathione S-transferase (GST), had higher CD4 cell count (β=3.48, p=0.034), lower HIV viral load (β=−0.536, p=0.018), and lower mitochondrial 8-oxo-dG (β=−0.28, p=0.03). ART reduced oxidative stress in the participants with the GSTM1 coding for the functional antioxidant enzyme. HIV/HCV co-infected participants with the GSTM1 coding for the functional antioxidant enzyme also had lower HIV viral load, lower 8-oxo-dG and lower rate of apoptosis, but also higher oxidized glutathione. Alcohol consumption was associated with lower HIV viral load but higher oxidized glutathione in those with the GSTM1 genotype coding for the functional antioxidant enzyme.

**Conclusion:**

The GSTM1 genotype coding for the functional antioxidant enzyme is associated with lower HIV disease severity, and with lower oxidative stress, compared to GSTM1 null-allele polymorphism. HCV co-infection and alcohol use may be associated with increased oxidative stress even in the presence of the GSTM1 coding for the functional antioxidant enzyme. The null-gene, on the contrary, appears to have a detrimental effect on immune function, viral load control, and antioxidant status, suggesting a potential benefit from antioxidants in HIV infected patients with the defective gene.

## Introduction

Mitochondrial damage is implicated in the etiology of complications of chronic human immunodeficiency virus (HIV) infection, as well as some adverse effects associated with antiretroviral therapy (ART) [1]. Mitochondrial dysfunction and accumulation of damaged mitochondrial DNA is also associated with several diseases of aging, including cancer [2] and is implicated in metabolic, renal, bone, and neurocognitive abnormalities occurring in patients with HIV [3–7]. Elevated oxidative stress increases the rate of mitochondrial DNA damage; in turn, accumulation of mitochondrial DNA (mtDNA) mutations further increases oxidative stress as well as cell apoptosis [2]. The HIV virus activates mitochondrial enzymes that lead to mitochondrial dysfunction and apoptosis of cells [8,9], including those of the immune system, such as CD4+ and CD8+ T cells [10]. Increased levels of markers of oxidative stress including oxidized glutathione, malondialdehyde (MDA), 8-oxo-2’-deoxyguanosine (8-oxo-dG), as well as the enzyme activity of electron transport chain enzymes Complex I and Complex IV [1,11–13], decreased antioxidants [14], and dysregulation of the mitochondrial transmembrane potential [15] have been observed in HIV infection and HIV/hepatitis C (HCV) co-infection [13–18]. This imbalance in the redox system has been associated with stimulation of viral replication via activation of nuclear factor κB and induction of apoptosis of CD4+ T-cells [13,19].

Glutathione-S-Transferases (GSTs) make up a family of phase II detoxification enzymes in the mitochondria and cytosol, responsible in part for response to oxidative stress in humans. The GST family includes a diverse range of enzymes; while they differ in their function, activity, and distribution, they all contribute to detoxification by catalyzing the oxidation of glutathione, a key intracellular antioxidant molecule [20]. Although, many genes code for GST enzymes, the gene GSTM1, which encodes a cytosolic GST of the *mu* subfamily, has been observed to be highly polymorphic [21]. A common polymorphism involving this gene prevents transcription of a functional copy of the *mu 1* GST enzyme. This GSTM1 “null allele,” when inherited homozygously, results in absent enzyme activity [22]. Recent studies have suggested that null genotype of GSTM1 may be associated with risk of cancer and other diseases related to oxidative stress; individuals with GSTM1 null allele polymorphism have been observed to be at greater risk for cancers of the breast, prostate, cervix, skin, and mouth, as well as atopic asthma [22–27].

Although deletion of GSTM1 has been identified as a risk factor for diseases of oxidative stress, and oxidative stress is implicated in the progression of HIV/AIDS, the effects of GSTM1 null allele polymorphism in HIV-infected populations are currently unknown. The primary objective of this investigation was to determine whether homozygous deletion of GSTM1 will affect oxidative stress levels and disease progression in HIV-infected and HIV/HCV co-infected individuals.

## Methods

### Study participants

A cohort of HIV infected participants, consecutively enrolled, was recruited to participate in this study in Miami, Florida, between February 2009 and August 2012. Participants were 40–60 years old, with CD4 cell count >350 cells/µl, and were HIV infected or HIV/HCV coinfected. Participants, who were HBV or HCV mono-infected, or HIV/ HBV co-infected, were excluded. By using a narrow age range, and CD4 count >350 cells/µl as an inclusion criteria, conditions previously found to be associated with elevated oxidative stress, i.e. aging [28] and low CD4 cell count [29], were excluded. The Institutional Review Board of the Florida International University (FIU) approved the study.

After providing consent, the participants were screened for eligibility, and underwent an assessment interview that included demographic, substance abuse and medical questionnaires. Weight and height were obtained in participants wearing light clothing and no shoes utilizing standard procedures. Body Mass Index (BMI) was calculated using the standard formula that divides the weight in Kg by the square of the height in meters (Kg/m2).

Physical examination was completed and anthropometries were measured at the Borinquen Family Healthcare Center/ FIU Research Clinic in Miami, Florida. HIV, HBV and HCV status, CD4+ cell count, and HIV viral load, were obtained from the participants’ medical charts with their consent. Fasting blood was drawn to determine genotypes for *GSTM1* allele polymorphism and to obtain markers of oxidative stress (percent plasma oxidized glutathione, peripheral blood mononuclear lymphocyte (PBMC) mitochondrial 8-oxo-dG, malondialdehyde (MDA), Complex I and IV enzyme activity, and apoptosis.

The genotypes for *GSTM1* null allele polymorphism were determined from DNA extracted from PBMC using QIAamp DNA mini kits (Qiagen; Valencia, CA). The presence or absence of the *GSTM1* allele was determined using quantitative PCR (qPCR) based on the protocol described by Rose-Zerilli et al. [30]. The assay consisted of Taqman minor groove-binding hydrolysis probes targeting both *GSTM1* and ALB (albumin) as a reference gene, performed in duplicate multiplex wells. Each 25 µl reaction volume contained 2 µl DNA at 5 ng/µl concentration, 12.5 µl 2X iQSupermix (Bio-Rad; Hercules, CA), GSTM1 primers (0.75 µl forward, 5’ GACTCTTGCATCCTGCACACA 3’, and 1.13 µl reverse, 5’ GGAAAGCACTTGGAGGATGAAT 3’), ALB primers (1.13 µl forward, 5’ CTGTCATCTCTTGTGGGCTGTAA 3’, and 0.75 µl reverse, 5’ GGCATGACAGGTTTTGCAATATT 3’), Taqman MGB probes (0.19 µl GSTM1, 6FAM-TGGTCTTAAGTCCCTGGTACMGB-NFQ, and 0.19 µl ALB, TET-CATCGTCTAGGCTTAAGAGMGB-NFQ), and PCR-grade water to achieve the proper volume. Thermal cycling was performed using an iQ5 qPCR machine (Bio-Rad; Hercules, CA). Samples underwent a thermal profile of 10 minutes incubation at 95°C, preceding 40 cycles of 15 seconds at 95°C and 60 seconds at 60°C. The amplification plots were analyzed manually to compare detection of the target sequence to the reference gene. Genotyping results were validated by inclusion of positive control samples from the ECACC Human Random Control DNA Panel (Sigma-Aldrich, St. Louis, MO).

Percent oxidized glutathione was determined with the Glutathione Colorimetric Detection Kit from Arbor Assays (Ann Arbor, MI), using an EL-800 micro-plate reader (Bio-Tek; Winooski, VT). The MDA protocol was performed with kits purchased from Northwest Life Science Specialties (Vancouver, WA) using a model DU-530 spectrophotometer by Beckman-Coulter, Inc. (Indianapolis, IN). Levels of mitochondrial 8-oxo-dG were determined by qPCR using a standard protocol measured with the iQ5 qPCR system. Complex I and IV activities were measured using an immuno-chromato-dipstick assay in an MS1000 immuno-chromato reader by Mitosciences Inc. (Eugene, OR). Epithelial cell apoptosis was measured by plasma levels of caspasecleaved cytokeratin (CK-18), determined by M30 Apoptosense ELISA kit (PEVIVA, Bromma, Sweden).

### Statistical analysis

All analyses were performed with SAS version 9.2, using a significance level of *P*=0.05. Descriptive statistics were used to characterize the population in terms of mean values for demographic and anthropometric variables, including sex, age, income, BMI, and waist/hip ratio. Analysis of Hardy-Weinberg equilibrium was not possible because the genotyping protocol was unable to differentiate GSTM1 as heterozygotes or wild-types. To normalize distribution of disease progression variables, HIV viral load was log transformed, and the square root was taken for CD4 cell count. Multivariate linear regression analyses were used to compare the binary variable of GSTM1 status with all continuous variables reflecting oxidative stress (percent oxidized glutathione, mitochondrial 8-oxo-dG, MDA, and Complex I and IV enzyme activity), apoptosis and disease progression (CD4 count and HIV viral load). These analyses were performed among the full sample, as well as between the two sub-cohorts (HIV/HCV co-infected individuals and alcohol users). Age- and gender-adjusted linear regression models were constructed to assess association of each of the following possible clinical correlates individually: CD4 cell count, HIV viral load, percent oxidized glutathione, MDA, mitochondrial 8-oxo-dG, Complex I and IV enzyme activity and apoptosis, compared the population with the GSTM1 genotype coding for the functional antioxidant enzyme with those with the null GSTM1 genotype. The parameters generated by these analyses include all available observations of each given variable among study participants belonging to the designated cohort or sub-cohort. The analyses for the oxidative stress were performed on values of individuals who had BMI <28 Kg/m^2^ and were adjusted for ART, liver disease, race, drug use and education. Separate analyses were performed with ART as an independent variable and HIV disease progression, oxidative stress and apoptosis as dependent variables to determine the contribution of ART on the effects of the GSTM1 genotype.

## Results

**Figure 1:**
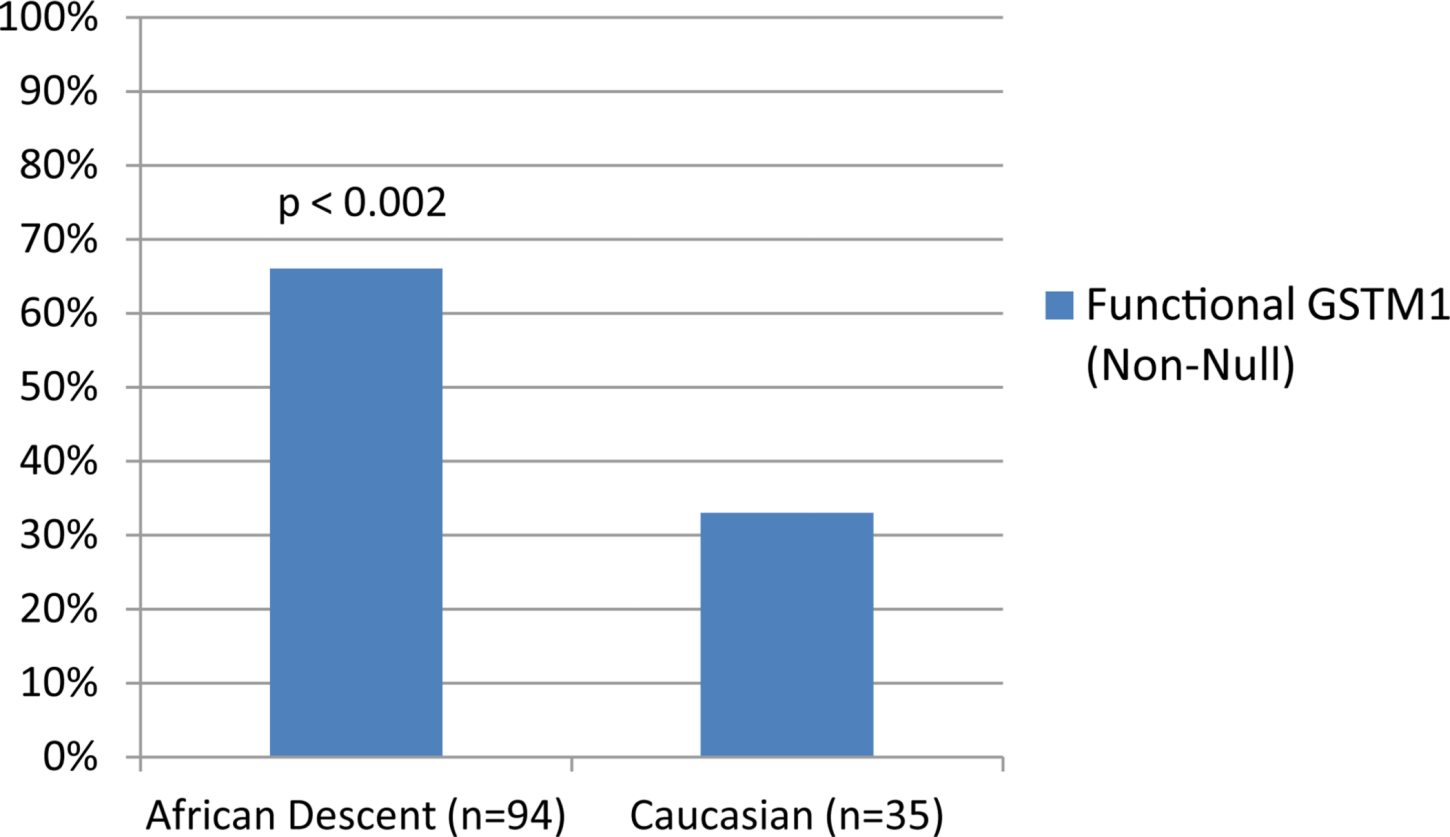
Frequency of the Functional (Non-Null) Genotype by Ethnicity.

There were a total of 129 HIV infected participants in the study, 58 (45%) were HIV/HCV co-infected; qPCR analysis identified 53 (41.2%) of the individuals as null GSTM1, and 76 (58.8%) with *GSTM1* coding for the functional antioxidant enzyme. The population characteristics of the study participants are shown in Table 1. The sample was 56% male and 44% female, with a mean age of 48.32 ± 6.31 years. Mean values for BMI and waist/hip ratio were 28.29 ± 5.29 Kg/m2 and 0.91 ± 0.07, respectively. Mean CD4 count was 591 ± 414 cells/µL and mean HIV viral load was 16,416 ± 94,636 copies/ml. Genotype frequency significantly differed based on race, as seen in Figure 1, among the 94 participants of African descent, 66% possessed the GSTM1 genotype coding for the functional antioxidant enzyme, while this genotype was observed in only 33% of the 35 Caucasian participants (p= 0.002).

There were no differences in other demographic variables between the participants who had the null vs. the *GSTM1* genotype that codes for the functional antioxidant enzyme.

Table 2 shows the mean and standard deviations for the biomarkers of HIV disease progression, and Table 3 the measures of oxidative stress associated with the GSTM1 genotype coding for the functional antioxidant enzyme when compared to those who had the null GSTM1 gene. T-test analyses showed that HIV viral load and the Complex I activity was significantly lower in the participants with the GSTM1 genotype coding for the functional antioxidant enzyme (not shown in the tables). When regression analysis was used to determine the effect of the GSTM1 status and the continuous variables reflecting HIV disease progression and oxidative stress, several significant relationships were revealed, as shown in Tables 2 and 3. Participants with the GSTM1 gene coding for the functional antioxidant enzyme had lower viral load (β=−0.536, p=0.018), and higher CD4 cell count (β=3.480, p=0.034), compared to those who had the null GSTM1 gene. Since obesity profoundly affects oxidative stress [31], only the values for participants with BMI <28 kg/m2 were used in the analyses when the biomarkers of oxidative stress were considered. Participants with the GSTM1 genotype coding for the functional antioxidant enzyme had lower levels of mitochondrial 8-oxo-dG (β=−0.28, p=0.030) than participants with the null GSTM1 genotype. When ART was used as an independent variable, those with the GSTM1 genotype coding for the functional antioxidant enzyme had lower MDA (β=−1.67, p=0.050), lower percent oxidized glutathione (β=−5.59, p=0.047), and lower Complex I (β=−13.83, p=0.040), but higher 8-oxo-dG (β=0.28, p=0.050, results not shown in the tables).

Tables 4 and 5 show the regression coefficients for the effect of the GSTM1 genotype on HIV disease progression and oxidative stress parameters in the two sub-cohorts. Participants with HIV/HCV co-infection with the GSTM1 genotype coding for the functional antioxidant enzyme (Table 4) had significantly lower HIV viral load (β=−0.918, p=0.022), lower mitochondrial 8-oxo-dG (β=−0.48, p=0.028), and lower rate of apoptosis (β=−67.40, p=0.009), but they also had higher oxidized glutathione (β=10.02, p=0.028) compared to those who had the null GSTM1 gene. The participants with the GSTM1 genotype coding for the functional antioxidant enzyme who consumed alcohol (Table 5) also had lower HIV viral load (β=−0.789, p=0.027), lower Complex I enzyme activity (β=−16.909, p=0.042), but significantly higher oxidized glutathione (β=10.63, p=0.029) compared to those who had the null GSTM1 gene. The GSTM1 genotype did not appear to affect the levels of MDA in any of the analyses.

In addition to the multivariable analyses to determine whether the *GSTM1* genotype affects markers of HIV disease progression and oxidative stress, we also conducted analyses to characterize the potential impact of the GSTM1 genotype on disease progression and oxidative stress among those who are HIV/HCV co-infected and those who consume alcohol. We compared HIV mono-infected and HIV/HCV co-infected participants and excluded those who drank alcohol (N=41). The analyses showed that the only variable that was significantly different between the two groups was hepatocyte apoptosis (β=46.15, *P*=0.015), which maintained significance after controlling for age, gender and the null *GSTM1* genotype (β=41.69, *P*=0.022). Comparison of HIV mono-infected participants who did or did not consume alcohol showed that the only variable that was significantly associated with alcohol consumption was hepatic apoptosis (β=33.43, *P*=0.049); this relationship however, lost significance when controlled for age, gender and the null *GSTM1* genotype showing that the *GSTM1* genotype was more important in determining apoptosis than alcohol consumption.

## Discussion

The results of this study indicate that the *GSTM1* genotype that codes for the functional antioxidant enzyme is not equally distributed by race, occurring in 33% of the Caucasians and 66% of the participants of African descent. The *GSTM1* genotype coding for the functional antioxidant enzyme was associated with more advantageous oxidative stress status, lower rate of apoptosis, and an apparent favorable effect on both immune function and viral load control in HIV-infected individuals. ART reduced oxidative stress in participants with the *GSTM1* genotype coding for the functional antioxidant enzyme. HCV status and alcohol use affected the association of the *GSTM1* genotype with oxidative stress, with both HCV co-infection and alcohol use increasing the levels of oxidative stress in this cohort.

Glutathione-S-transferases (GSTs) play a role in the detoxification of the reactive oxygen species [20]. The GSTM1 null-allele polymorphism is associated with reduced mitochondrial enzyme activity, decreased ability to detoxify compounds, increased level of reactive oxygen species, and increased risk of cancers [32–34], diabetes mellitus [35], and other diseases of aging [2,23–27], primarily among Caucasians [34]. While individual glutathione-S-tranferase polymorphisms influence vulnerability to oxidative stress, *GSTM1*-null gene variant has the most pronounced effect [36]. Only one study was conducted in an HIV infected population to the best of our knowledge to date, investigating a decreased response to ART in smoking HIV infected women. This study, however, showed that *GSTM1* deletions were not associated with the effectiveness of the treatment in smokers [37]. Increased levels of oxidative stress in HIV infected patients, relative to healthy subjects, have been demonstrated [13–18]. However, the possible effect of the *GSTM1* genotype has not been investigated in association with the progression of HIV disease or oxidative stress variables.

Our results show that the GSTM1 gene coding for the functional antioxidant enzyme appears to play a role in the defense system against HIV infection, since HIV viral load was significantly and consistently lower in participants with the GSTM1 genotype that codes for the functional antioxidant enzyme. Moreover, the lower levels of HIV viral load were independent of the higher oxidative stress found in HIV/ HCV co-infected participants, and in those who consumed alcohol. In addition, CD4 count was significantly higher in the participants with the GSTM1 genotype coding for the functional antioxidant enzyme. The mechanisms of how the functional antioxidant enzyme confers better protection for HIV disease severity is not known at this time; our studies with antioxidants [18,38,39] and those of others [40,41] indicate that, while the primary effect may be on the viral burden, the observed benefit on CD4 cell count is consistent, with previous studies showing a direct relationship between serum antioxidants and CD4 cell count [42,43], opportunistic infections, disease progression, and HIV-related mortality [44–46]. In addition, there appears to be a disadvantage of reduced detoxification in GSTM1-null individuals that may promote HIV disease progression.

Alterations of the level of oxidative stress or the redox balance of cells may have major implications for normal function of cells, and therefore, it is important to consider each biomarker of oxidative stress in order to determine the overall relationship of GSTM1 null-allele polymorphism. Since a number of studies have shown associations between increased oxidative stress, obesity [47,48], insulin resistance [49], and liver disease [50,51], only the values for participants without diabetes, whose BMI was <28 kg/m2, and adjusted for liver disease, were used in the analysis considering oxidative stress. Increased levels of mitochondrial 8-oxo-dG reflect the effect of increased oxidative stress on mitochondrial DNA, with 8-oxo-dG as one of the major products of mitochondrial DNA oxidation [1]. Thus, the significantly lower levels of mitochondrial 8-oxo-dG in participants with GSTM1 genotype coding for the functional antioxidant enzyme indicate the important advantage conferred in the defense against DNA damage. The reduced oxidative stress levels associated with lower 8-oxo-dG may contribute to the favorable effects on disease progression experienced by individuals with GSTM1 coding for the functional antioxidant enzyme. The observed lack of association of GSTM1 genotype with MDA, a product of oxidative damage resulting in lipid peroxidation, is consistent with reported results in other conditions, including diabetes [52,53], renal disease [36], and coronary artery disease [54], suggesting that this polymorphism has an insignificant effect on lipid peroxidation in chronic disease states.

Glutathione, an antioxidant found in all cells, prevents damage caused by oxidative stress; levels of oxidized glutathione reflect oxidative damage associated with chronic inflammatory response in HIV infected populations [13]. The results of this study showed increased percent oxidized glutathione in the HIV/HCV co-infected cohort and in participants who consumed alcohol in the presence of lower 8-oxodG, and apoptosis indicating lower oxidative stress in participants with

GSTM1 coding for the functional antioxidant enzyme (Tables 4 and 5). It is unexpected that the oxidized glutathione would be unaffected by *GSTM1* polymorphism when 8-oxo-dG levels are reduced. A possible explanation for this finding lies in cooperation of enzymes contributing to the cellular redox system. Individuals with null genotype of *GSTM1* have increased concentrations of compensatory antioxidant enzymes after exposure to oxidative stress; in particular, activity of superoxide dismutase has been reported to be significantly higher in individuals with null vs. GSTM1 gene after exposure to ozone [55]. Further research is needed to confirm whether compensatory up regulation of this nature could be a factor in the antioxidant status of individuals with null GSTM1 gene.

Apart from the effect of HIV, other variables also contribute significantly to oxidative stress in HIV-infected individuals, including co-infection with hepatitis C and alcohol use. Dual infection of HIV and HCV has become a leading cause of mortality among HIV infected patients, and has been associated with increased levels of oxidative stress [18,56]. Alcohol use generates oxidative damage in pathways similar to those associated with HIV, leading to a greater combined effect than either risk factor alone [57]. The analysis of the HIV/HCV co-infected and alcohol-drinking sub-cohorts in this study revealed similar effects on oxidative stress and disease progression, with some notable exceptions. Although HIV viral load remained significantly lower in individuals with the non-null *GSTM1* vs. the null gene in both sub-cohorts, neither group had significant genotype association with CD4 cell count. This was likely caused by the smaller sample size of the sub-cohort groups, which no longer had sufficient power to examine the relationship. Another novel finding of the sub-cohorts compared to the full sample was the elevated levels of oxidized glutathione observed in the HIV/HCV co-infected and those who consumed alcohol with the *GSTM1* genotype status coding for the functional antioxidant enzyme. These results may indicate the proposed compensatory upregulation of coexisting antioxidant enzymes.

Activities of electron transport chain enzymes Complex I and IV are distinct indicators of mitochondrial toxicity and dysfunction of oxidative phosphorylation [58]. The lower Complex I enzyme activity along with significantly lower apoptosis in the HIV/HCV co-infected participants with the GSTM1 genotype coding for the functional antioxidant enzyme are consistent with the reports of differential regulation of the two complexes in times of acute HIV infection and induction of apoptosis [59]. This suggests that those individuals possessing the null mutation may be more susceptible to dysregulation of oxidative phosphorylation by HIV, and ultimately T-cell apoptosis, compared to those with the GSTM1 coding for the functional antioxidant enzyme.

Analyses to characterize the potential impact of the GSTM1 genotype on disease progression and oxidative stress among those who are HIV/HCV co-infected and those who consumed alcohol revealed modulated effects of the GSTM1 null-allele polymorphism. While both HIV/HCV co-infection and alcohol consumption increased hepatic apoptosis, having the GSTM1 null genotype increased oxidative stress particularly for alcohol consumption.

The findings of this study show that null-allele polymorphism of GSTM1 has a significant effect on oxidative stress and antioxidant status during HIV infection. Furthermore, this effect is clinically detectable in its impact on CD4 cell count and HIV viral load. This investigation represents only an initial assessment of the polymorphism’s role in HIV infection, and further research will be critical to discerning the mechanism involved and any potential therapeutic applications.

A comparison of the activities of antioxidant enzymes coexisting alongside GSTs, in relation to the GSTM1 genotype, will be needed to elucidate variation in levels of oxidative stress biomarkers. Additionally, genotyping techniques with the ability to differentiate between individuals carrying one or two copies of the GSTM1 gene would allow for more detailed analysis of the polymorphism’s effects. If the role of this polymorphism in HIV infection is further clarified, there is potential for GSTM1 to be used as a clinical marker to quantify risk for oxidative damage in HIV-infected individuals, possibly serving as a criterion to demonstrate a need for antioxidant supplementation. Further research will be needed to explore these possibilities, and to unfold the pathways that control oxidative stress and HIV disease progression.

## Figures and Tables

**Table 1:** Population Characteristics.

Characteristic	Overall (*n* = 129) [mean ± SD]	GSTM1 Null (*n* = 53) [mean ± SD]	GSTM1 Non-Null (*n* = 76) [mean ± SD]	P-value
Male [% (*n*)]	55.81% (72)	52.83% (28)	57.89% (44)	0.572
Age (years)	48.32 ± 6.31	48.53 ± 6.78	48.17 ± 6.00	0.755
CD4 count (cells/uL)	591 ± 414	592 ± 505	590 ± 295	0.988
HIV viral load (copies/mL)	16,416 ± 94,636	34,633 ± 149,550	4,769 ± 16,098	0.222
Monthly Income ($)	469.33 ± 484.33	450.10 ± 494.60	482.30 ± 480.90	0.741
Education (grades)	11.76 ± 2.15	11.70 ± 2.05	11.81 ± 2.23	0.809
BMI (kg/m^2^)	28.29 ± 5.29	28.99 ± 5.22	27.81 ± 5.31	0.217
Waist/Hip Ratio	0.91 ± 0.07	0.92 ± 0.07	0.91 ± 0.07	0.651
HIV/HCV co-infected [%(*n*)]	44.96% (58)	50.94% (27)	40.79% (31)	0.258
Receiving ART [%(*n*)]	89.92% (116)	92.45% (49)	88.16% (67)	0.429

**Table 2:** Effects of the Functional (Non-Null) GSTM1 Genotype on HIV Disease Progression.

VariableMean ± SD	Parameter Estimate	Standard Error	T-value	P-value
HIV viral load (copies/mL) 16,416 ± 94,636	−0.536	0.223	−2.41	0.018*
CD4 cell count (cells/uL) 591 ± 414	3.480	1.618	2.15	0.034*

* Indicates statistically significant results. P-value associated with linear regression analysis.

**Table 3:** Effects of the Functional (Non-Null) GSTM1 Genotype on Markers of Oxidative Stress.

Variable	Mean ± SD	Parameter Estimate	Standard Error	T-value	P-value
Oxidized glutathione(%)	24.57 ± 8.28	1.088	1.756	0.62	0.538
MDA (uM)	0.58 ± 0.22	0.038	0.049	0.77	0.443
Mitochondrial 8-oxo-dG(ΔCt)	0.53 ± 0.45	−0.277	0.123	−2.25	0.030*
Complex I enzyme activity (%)	47.81 ± 18.48	−4.984	5.226	−0.95	0.345
Complex IV enzyme activity (%)	25.43 ± 18.11	0.790	4.042	0.20	0.846
Apoptosis (CK18 Average value (pM)	127.18 ± 76.75	−18.350	18.976	−0.97	0.338

* Indicates statistically significant results. P-value associated with linear regression analysis. Participants with BMI>28 kg/m^2^ excluded from regression analysis.

**Table 4:** Effects of the Functional (Non-Null) GSTM1 Genotype on HIV Disease Progression and Oxidative Stress in HIV/HCV Co-Infected Sub-Cohort.

Variable	Sub-Cohort Mean ± SD (*n*= 58)	Parameter Estimate	Standard Error	T-value	P-value
HIV viral load (copies/mL)^A^	29,058±137,990	−0.918	0.385	−2.39	0.022*
CD4 cell count (cells/uL)^A^	557 ± 355	1.236	2.467	0.50	0.619
Oxidized glutathione(%)	26.49 ± 10.43	10.021	4.156	2.41	0.028*
MDA (uM)	0.54 ± 0.25	0.030	0.087	0.34	0.736
Mitochondrial 8-oxo-dG (ΔCt)	0.44 ± 0.53	−0.478	0.201	−2.38	0.028*
Complex I enzyme activity (%)	46.85 ± 16.36	−0.216	7.593	−0.03	0.978
Complex IV enzyme activity (%)	21.96 ± 14.65	−4.946	4.093	−1.21	0.246
Apoptosis (CK18 Average value (pM)^B^	130.49 ± 80.47	−67.489	22.453	−3.01	0.009*

* Indicates statistically significant results. P-value associated with linear regression analysis. Participants with BMI>28 kg/m^2^ excluded from regression analysis.

A No exclusion of participants with BMI>28 in this analysis.

B Participants who consume alcohol excluded.

**Table 5:** Effects of the Functional (Non-Null) GSTM1 Genotype on HIV Disease Progression and Oxidative Stress in Alcohol-Drinking Sub-Cohort.

Variable	Sub-Cohort Mean ± SD (*n*= 58)	Parameter Estimate	Standard Error	T-value	P-value
HIV viral load (copies/mL)^A^	7,856 ± 27,585	−0.789	0.342	−2.31	0.027*
CD4 cell count (cells/uL)^A^	483 ± 279	36.195	97.733	0.37	0.713
Oxidized glutathione (%)	25.77 ± 9.96	10.625	4.292	2.48	0.029*
MDA (uM)	0.55 ± 0.23	0.098	0.095	1.04	0.311
Mitochondrial 8-oxo-dG (ΔCt)	0.57 ± 0.48	0.009	0.181	0.05	0.961
Complex I enzyme activity (%)	43.84 ± 20.11	−16.909	7.624	−2.22	0.042*
Complex IV enzyme activity (%)	22.09 ± 16.44	1.798	10.238	0.18	0.863
Apoptosis (CK18 Average value (pM))	126.91 ± 61.15	−5.070	25.903	−0.20	0.847

* Indicates statistically significant results. P-value associated with linear regression analysis. Participants with BMI>28 kg/m^2^ excluded from regression analysis unless otherwise specified

A No exclusion of participants with BMI>28 in this analysis.

## References

[1] Hulgan T, Gerschenson M (2012). HIV and mitochondria: more than just drug toxicity. J Infect Dis.

[2] Wallace DC (2005). A mitochondrial paradigm of metabolic and degenerative diseases, aging, and cancer: a dawn for evolutionary medicine. Annu Rev Genet.

[3] Feeney ER, Mallon PW (2010). Impact of mitochondrial toxicity of HIV-1 antiretroviral drugs on lipodystrophy and metabolic dysregulation. Curr Pharm Des.

[4] Grinspoon S, Carr A (2005). Cardiovascular risk and body-fat abnormalities in HIV-infected adults. N Engl J Med.

[5] Rodriguez-Nóvoa S, Alvarez E, Labarga P, Soriano V (2010). Renal toxicity associated with tenofovir use. Expert Opin Drug Saf.

[6] McComsey GA, Tebas P, Shane E, Yin MT, Overton ET (2010). Bone disease in HIV infection: a practical review and recommendations for HIV care providers. Clin Infect Dis.

[7] Heaton RK, Clifford DB, Franklin DR, Woods SP, Ake C (2010). HIV-associated neurocognitive disorders persist in the era of potent antiretroviral therapy: CHARTER Study. Neurology.

[8] Roumier T, Castedo M, Perfettini JL, Andreau K, Métivier D (2003). Mitochondrion-dependent caspase activation by the HIV-1 envelope. Biochem Pharmacol.

[9] Muthumani K, Choo AY, Hwang DS, Chattergoon MA, Dayes NN (2003). Mechanism of HIV-1 viral protein R-induced apoptosis. Biochem Biophys Res Commun.

[10] Arnoult D, Petit F, Lelièvre JD, Estaquier J (2003). Mitochondria in HIV-1-induced apoptosis. Biochem Biophys Res Commun.

[11] Muñoz JF, Salmen S, Berrueta LR, Carlos MP, Cova JA (1999). Effect of human immunodeficiency virus type 1 on intracellular activation and superoxide production by neutrophils. J Infect Dis.

[12] Olivetta E, Pietraforte D, Schiavoni I, Minetti M, Federico M (2005). HIV-1 Nef regulates the release of superoxide anions from human macrophages. Biochem J.

[13] Gil L, Martínez G, González I, Tarinas A, Alvarez A (2003). Contribution to characterization of oxidative stress in HIV/AIDS patients. Pharmacol Res.

[14] Morris D, Guerra C, Donohue C, Oh H, Khurasany M (2012). Unveiling the mechanisms for decreased glutathione in individuals with HIV infection. Clin Dev Immunol.

[15] Perl A, Banki K (2000). Genetic and metabolic control of the mitochondrial transmembrane potential and reactive oxygen intermediate production in HIV disease. Antioxid Redox Signal.

[16] Repetto M, Reides C, Gomez Carretero ML, Costa M, Griemberg G (1996). Oxidative stress in blood of HIV infected patients. Clin Chim Acta.

[17] Baum MK (2000). Role of micronutrients in HIV-infected intravenous drug users. J Acquir Immune Defic Syndr.

[18] Baum MK, Sales S, Jayaweera DT, Lai S, Bradwin G (2011). Coinfection with hepatitis C virus, oxidative stress and antioxidant status in HIV-positive drug users in Miami. HIV Med.

[19] Israël N, Gougerot-Pocidalo MA (1997). Oxidative stress in human immunodeficiency virus infection. Cell Mol Life Sci.

[20] Ketterer B (2001). A bird's eye view of the glutathione transferase field. Chem Biol Interact.

[21] Moyer AM, Salavaggione OE, Hebbring SJ, Moon I, Hildebrandt MA (2007). Glutathione S-transferase T1 and M1: gene sequence variation and functional genomics. Clin Cancer Res.

[22] Rebbeck TR (1997). Molecular epidemiology of the human glutathione S-transferase genotypes GSTM1 and GSTT1 in cancer susceptibility. Cancer Epidemiol Biomarkers Prev.

[23] Anantharaman D, Samant TA, Sen S, Mahimkar MB (2011). Polymorphisms in tobacco metabolism and DNA repair genes modulate oral precancer and cancer risk. Oral Oncol.

[24] Gao LB, Pan XM, Li LJ, Liang WB, Bai P (2011). Null genotypes of GSTM1 and GSTT1 contribute to risk of cervical neoplasia: an evidence-based meta-analysis. PLoS One.

[25] Lear JT, Heagerty AH, Smith A, Bowers B, Payne CR (1996). Multiple cutaneous basal cell carcinomas: glutathione S-transferase (GSTM1, GSTT1) and cytochrome P450 (CYP2D6, CYP1A1) polymorphisms influence tumors numbers and accrual. Carcinogenesis.

[26] Taioli E, Flores-Obando RE, Agalliu I, Blanchet P, Bunker CH (2011). Multi-institutional prostate cancer study of genetic susceptibility in populations of African descent. Carcinogenesis.

[27] Brasch-Andersen C, Christiansen L, Tan Q, Haagerup A, Vestbo J (2004). Possible gene dosage effect of glutathione-S-transferases on atopic asthma: using real-time PCR for quantification of GSTM1 and GSTT1 gene copy numbers. Hum Mutat.

[28] Wang CH, Wu SB, Wu YT, Wei YH (2013). Oxidative stress response elicited by mitochondrial dysfunction: Implication in the pathophysiology of aging. Exp Biol Med (Maywood).

[29] Glesby MJ, Hoover DR, Raiszadeh F, Lee I, Shi Q (2009). Oxidant stress in HIV-infected women from the Women's Interagency HIV Study. Antivir Ther.

[30] Rose-Zerilli MJ, Barton SJ, Henderson AJ, Shaheen SO, Holloway JW (2009). Copy-number variation genotyping of GSTT1 and GSTM1 gene deletions by real-time PCR. Clin Chem.

[31] Razavi A, Baghshani MR, Ardabili HM, Andalibi MS, Rahsepar AA (2013). Obese subjects have significantly higher serum prooxidantantioxidant balance values compared to normal-weight subjects. Clin Lab.

[32] Liu D, Liu Y, Ran L, Shang H, Li D (2013). GSTT1 and GSTM1 polymorphisms and prostate cancer risk in Asians: a systematic review and meta-analysis. Tumour Biol.

[33] Wang D, Wang B, Zhai JX, Liu DW, Sun GG (2011). Glutathione S-transferase M1 and T1 polymorphisms and cervical cancer risk: a meta-analysis. Neoplasma.

[34] Economopoulos KP, Sergentanis TN (2010). GSTM1, GSTT1, GSTP1, GSTA1 and colorectal cancer risk: a comprehensive meta-analysis. Eur J Cancer.

[35] Zhang J, Liu H, Yan H, Huang G, Wang B (2013). Null genotypes of GSTM1 and GSTT1 contribute to increased risk of diabetes mellitus: a meta-analysis. Gene.

[36] Suvakov S, Damjanovic T, Stefanovic A, Pekmezovic T, Savic-Radojevic A (2013). Glutathione S-transferase A1, M1, P1 and T1 null or low-activity genotypes are associated with enhanced oxidative damage among haemodialysis patients. Nephrol Dial Transplant.

[37] Feldman DN, Feldman JG, Greenblatt R, Anastos K, Pearce L (2009). CYP1A1 genotype modifies the impact of smoking on effectiveness of HAART among women. AIDS Educ Prev.

[38] Hurwitz BE, Klaus JR, Llabre MM, Gonzalez A, Lawrence PJ (2007). Suppression of human immunodeficiency virus type 1 viral load with selenium supplementation: a randomized controlled trial. Arch Intern Med.

[39] Baum MK, Lai S, Sales S, Page JB, Campa A (2010). Randomized, controlled clinical trial of zinc supplementation to prevent immunological failure in HIV-infected adults. Clin Infect Dis.

[40] Winklhofer-Roob BM, Rock E, Ribalta J, Shmerling DH, Roob JM (2003). Effects of vitamin E and carotenoid status on oxidative stress in health and disease. Evidence obtained from human intervention studies. Mol Aspects Med.

[41] Bunupuradah T, Ubolyam S, Hansudewechakul R, Kosalaraksa P, Ngampiyaskul C (2012). Correlation of selenium and zinc levels to antiretroviral treatment outcomes in Thai HIV-infected children without severe HIV symptoms. Eur J Clin Nutr.

[42] Look MP, Rockstroh JK, Rao GS, Kreuzer KA, Spengler U (1997). Serum selenium versus lymphocyte subsets and markers of disease progression and inflammatory response in human immunodeficiency virus-1 infection. Biol Trace Elem Res.

[43] Cirelli A, Ciardi M, de Simone C, Sorice F, Giordano R (1991). Serum selenium concentration and disease progress in patients with HIV infection. Clin Biochem.

[44] Constans J, Pellegrin JL, Sergeant C, Simonoff M, Pellegrin I (1995). Serum selenium predicts outcome in HIV infection. J Acquir Immune Defic Syndr Hum Retrovirol.

[45] Baum MK, Shor-Posner G, Lai S, Zhang G, Lai H (1997). High risk of HIV-related mortality is associated with selenium deficiency. J Acquir Immune Defic Syndr Hum Retrovirol.

[46] Campa A, Shor-Posner G, Indacochea F, Zhang G, Lai H (1999). Mortality risk in selenium-deficient HIV-positive children. J Acquir Immune Defic Syndr Hum Retrovirol.

[47] Keaney JF, Larson MG, Vasan RS, Wilson PW, Lipinska I (2003). Obesity and systemic oxidative stress: clinical correlates of oxidative stress in the Framingham Study. Arterioscler Thromb Vasc Biol.

[48] Urakawa H, Katsuki A, Sumida Y, Gabazza EC, Murashima S (2003). Oxidative stress is associated with adiposity and insulin resistance in men. J Clin Endocrinol Metab.

[49] Hirashima O, Kawano H, Motoyama T, Hirai N, Ohgushi M (2000). Improvement of endothelial function and insulin sensitivity with vitamin C in patients with coronary spastic angina: possible role of reactive oxygen species. J Am Coll Cardiol.

[50] Mehta K, Van Thiel DH, Shah N, Mobarhan S (2002). Nonalcoholic fatty liver disease: pathogenesis and the role of antioxidants. Nutr Rev.

[51] Bujanda L, Hijona E, Larzabal M, Beraza M, Aldazabal P (2008). Resveratrol inhibits nonalcoholic fatty liver disease in rats. BMC Gastroenterol.

[52] Hayek T, Stephens JW, Hubbart CS, Acharya J, Caslake MJ (2006). A common variant in the glutathione S transferase gene is associated with elevated markers of inflammation and lipid peroxidation in subjects with diabetes mellitus. Atherosclerosis.

[53] Datta SK, Kumar V, Ahmed RS, Tripathi AK, Kalra OP (2010). Effect of GSTM1 and GSTT1 double deletions in the development of oxidative stress in diabetic nephropathy patients. Indian J Biochem Biophys.

[54] Ueno T, Watanabe H, Fukuda N, Tsunemi A, Tahira K (2009). Influence of genetic polymorphisms in oxidative stress related genes and smoking on plasma MDA-LDL, soluble CD40 ligand, E-selectin and soluble ICAM1 levels in patients with coronary artery disease. Med Sci Monit.

[55] Otto-Knapp R, Jurgovsky K, Schierhorn K, Kunkel G (2003). Antioxidative enzymes in human nasal mucosa after exposure to ozone. Possible role of GSTM1 deficiency. Inflamm Res.

[56] Monga HK, Rodriguez-Barradas MC, Breaux K, Khattak K, Troisi CL (2001). Hepatitis C virus infection-related morbidity and mortality among patients with human immunodeficiency virus infection. Clin Infect Dis.

[57] Persidsky Y, Ho W, Ramirez SH, Potula R, Abood ME (2011). HIV-1 infection and alcohol abuse: neurocognitive impairment, mechanisms of neurodegeneration and therapeutic interventions. Brain Behav Immun.

[58] Fosslien E (2001). Mitochondrial medicine--molecular pathology of defective oxidative phosphorylation. Ann Clin Lab Sci.

[59] Tripathy MK, Mitra D (2010). Differential modulation of mitochondrial OXPHOS system during HIV-1 induced T-cell apoptosis: up regulation of Complex-IV subunit COX-II and its possible implications. Apoptosis.

